# Stability of serum ferritin measured by immunoturbidimetric assay after storage at -80°C for several years

**DOI:** 10.1371/journal.pone.0188332

**Published:** 2017-12-11

**Authors:** Anne-Sylvia Sacri, Daniela Ferreira, Babak Khoshnood, Laurent Gouya, Henrique Barros, Martin Chalumeau

**Affiliations:** 1 INSERM UMR1153, Centre de Recherche en Épidémiologie et Statistique Sorbonne Paris Cité (CRESS), équipe Épidémiologie Périnatale, Obstétricale et Pédiatrique (ÉPOPé); Labex GR-Ex; Université Paris Descartes; Paris, France; 2 Department of General Pediatrics and Pediatric Infectious Diseases, Necker-Enfants malades hospital, AP-HP; Université Paris Descartes, Paris, France; 3 Université Paris Diderot, Sorbonne Paris Cité, Paris, France; 4 EPIUnit—Instituto de Saúde Pública, Universidade do Porto, Porto, Portugal; 5 Centre de recherche sur l'inflammation, INSERM UMR 1149, Université Paris Diderot; ERL CNRS 8252, Faculté de Médecine site Bichat, Labex GR-Ex, Paris, France; Pennsylvania State University College of Medicine, UNITED STATES

## Abstract

**Background:**

Iron deficiency (ID) may impair long-term neurological development when it occurs in young infants. In cohort studies, it is sometimes necessary to evaluate ID with sera kept frozen for several years. To assess ID, learned societies recommend measuring serum ferritin (SF) level combined with C-reactive protein level. The long-term stability of C-reactive protein in frozen samples is well established but not ferritin.

**Methods:**

We measured SF level (immunoturbidimetric assay; in micrograms per liter) immediately after collection from 53 young adults recruited and followed-up in Porto, Portugal, from 2011 to 2013 (SF_1_), and then, in 2016 in two aliquots kept frozen at– 80°C for 3 to 5 years: one without (SF_2A_) and one with (SF_2B_) intermediate thawing in 2014. We compared SF_1_ to SF_2A_ then SF_2B_; statistical agreement was evaluated by the Bland and Altman method and the effect of intermediate thawing by regression modelling.

**Results:**

Mean SF_2A_–SF_1_ and SF_2B_–SF_1_ differences were -2.1 (SD 7.0) and 48.9 (SD 66.9). Values for Bland and Altman 95% limits of agreement were higher for the comparison of SF_2B_ and SF_1_ than SF_2A_ and SF_1_: -82.2 to 179.9 and -15.8 to 11.8, respectively; the effect of thawing was highly significant (p <0.001).

**Conclusions:**

Agreement between SF values before and after 3 to 5 years of constant freezing at -80°C was in a generally accepted range, which supports the hypothesis of ferritin’s stability at this temperature for a long period. In long-term storage by freezing, intermediate thawing induced a major increase in values.

## Introduction

Iron deficiency (ID) is considered the most frequent micronutrient deficiency worldwide, including in industrialized countries [1], and is suspected to be associated with adverse short- and long-term neurocognitive sequelae when it occurs in young people [2–5]. ID is a target of various primary prevention strategies and the subject of many studies [6–9]. In cohort studies, including birth ones, it is sometimes necessary to evaluate ID with sera stored for several years, with or without intermediate thawing. The World Health Organization, American Academy of Pediatrics and European Food Safety Authority recommend using serum ferritin (SF) level to measure ID [1, 6, 9]. SF well reflects body iron stores [10], and its level decreases in the first “pre-latent” stage of ID, which involves a reduction in the body's iron stores without any impairment of erythropoiesis [8, 10]. SF level increases with inflammation, and recommendations suggest coupling its measurement with that of C-reactive protein (CRP), an acute-phase inflammation protein [9, 11].

Comparability of CRP levels after freezing (-80°C to -70°C) during long periods (7 to 14 years [12–14]) is well established, but few data are available for SF levels. SF levels reported after storage at positive temperature varies widely during short periods (from 4 hrs to 2 weeks) [15–17] but SF comparability is considered acceptable after storage at -20°C, -70°C, -80°C and -196°C for 4 days to 12 months [18–20] (**S1 Table**). A few studies have compared SF levels after a freezing–thawing procedure or after a storage at positive temperature during long periods to those after immediate freezing. They reported comparable SF levels measures except if stored at +32°C [21–23]. However, in these studies, the time interval between the two measures was 13 months maximum, a short period compared to the times commonly encountered in birth-cohort or large-scale cohort studies [24]. Only one study compared SF levels after storage at -25°C during 2 years and 25 years with 1-month-old samples [25] and found notable percentage differences in mean SF level values: -12.1% and -18.5% after 2 and 25 years, respectively. Such alarming results could discourage the use of ferritin to evaluate ID in sera kept frozen on the long-term (>12 months). However, the data were obtained from non-paired samples. Furthermore, no data have been published on long-term stability at -80°C nor on the effect of intermediate thawing, which may occur during long storage. Finally, in studies evaluating the long-term stability of ferritin, comparability was assessed by correlation, coefficient of variation (CV) or mean differences and rarely with recommended agreement measures such as the Bland and Altman method [26].

The objective of this work was to evaluate the comparability of SF levels after storage for several years by freezing at -80°C and the impact of intermediate thawing on these conditions, using recommended agreement evaluation methods.

## Methods

### General methodology and participants

The present study is an ancillary analysis of an adolescent cohort (Epidemiological Health Investigation of Teenagers in Porto [EPITeen]) performed in Porto, Portugal in 2003–2004 [27, 28]. It is based on frozen serum samples from 53 participants who were young adults in 2011–2013. Parents of included adolescents gave their written consent after receiving information from the investigator, and written informed consent was obtained from all adult participants. The study was approved by the Ethics Committee of the University Hospital of São João (Porto, Portugal).

### Samples and measurements

For the present ancillary study, serum samples from the initial population in EPITeen were selected after considering the following criteria regarding the participants and the samples: a measurement of SF performed at the 2011–2013 follow-up and at least four frozen aliquots kept at -80°C. Then, samples were randomly selected among each of five groups of the initial SF levels—<10; 10–29, 30–99, 100–199, and ≥200 μg/L—to allow for representation of extreme values. SF levels were measured by immunoturbidimetric assay (Beckman Coulter, Krefeld, Germany) and were reported in micrograms per liter. According to the manufacturer’s information, the ferritin procedure is linear from 8 to 450 μg/L with recovery within 10% or 3 μg/L; the “within-run” precision and “total” precision SD are 1.15 to 1.89 and 1.21 to 4.36, respectively [29]; the limit of detection is < 4.6 μg/L and limit of quantification < 7.8 μg/L. In 2011–2013, blood samples were collected in VACUMED 16x100 mm tubes with an inert separator gel and a clot activator and centrifuged within 2 hrs after collection. Several serum aliquots were separated for immediate storage at -80°C (including aliquots A and B), and one aliquot (named 1) was immediately sent to the laboratory for analysis, as defined by the project protocol. With this aliquot 1, SF level was measured within 6 hrs after blood collection (SF_1_).

For the present study (**S1 Fig**), we selected a sample of aliquots, one that had not been frozen corresponding to aliquots 1 (n = 53), then the matched sample of aliquots A, frozen constantly until 2016 (n = 53), and another matched sample of aliquots B that had undergone intermediate thawing within 24 hrs, in 2014 (n = 28). This thawing was justified by the need for another laboratory assay and was followed by a new cycle of storage at -80°C that took place no longer than 24 hrs after thawing. Finally, in 2016, after 3 to 5 years of storage, aliquots A and B were thawed on the day of the current assay, at ambient temperature, before a second measure of SF (subsequently named SF_2A_ and SF_2B_ for aliquots A and B) immediately performed with the same method as for SF_1_.

### Statistical analyses

First, we analyzed the comparability of SF levels before and after long-term freezing without intermediate thawing by describing SF_1_ and SF_2A_ (n = 53 pairs); calculating their mean difference (SF_2A_–SF_1_), its SD, and the CV between SF_2A_ and SF_1_; and by building an equality line graph. SF_1_ and SF_2A_ distributions were compared by Student *t* test for paired samples. Agreement was evaluated by calculating the intraclass correlation coefficient (ICC) in a two-way mixed-effect model [30] and by the Bland and Altman method (calculating limits of agreement [LOA] with a Bland-Altman difference plot, as recommended [26]). Then, we compared the proportion of ID among SF_1_ and SF_2A_ distributions after dichotomization around the classical 15-μg/L threshold [1] by using the McNemar test for paired samples.

Second, we analyzed the comparability of SF levels before and after long-term freezing with intermediate thawing by comparing SF_1_ and SF_2B_ (n = 28 pairs) using the same statistical approach described above.

Third, the quantitative effect of thawing on the overall comparability of SF levels after long-term freezing was evaluated by using a linear regression model after testing the deviance to linearity. For this, we defined a new variable, SF_2_, that corresponded to SF_2B_ if available (n = 28 sera) or to SF_2A_ otherwise (n = 25 sera). We built a linear regression model with SF_1_ as the dependent variable and the independent variables SF_2_, thawing (binary variable “yes/no”) and an interaction term between “thawing” and SF_2_. The ICC between SF_1_, SF_2A_ and SF_2B_ in a two-way mixed-effect model was also calculated for the 28 participants who had 3 SF level measures available.

The analyses involved use of Stata/SE 13.1 (StataCorp, USA).

### Post-hoc supplementary analyses

Given the results on the effect of thawing in the case of long-term storage by freezing, we performed supplementary experiments to explore its effect during short-term storage by freezing. We conducted the experiment on a convenience sample of 6 participants. SF dosages were performed in the same laboratory and with the same techniques as the main study. For each participant, 4 SF dosages were made on three aliquots: one immediately after blood sampling (SF_A_), one after continuous freezing at -80°C during 7 days (SF_B_) and two on the same aliquot on day 3 (SF_C_) and day 7 (SF_D_) after freezing at -80°C with an intermediate thawing at day 3. We performed similar statistical analyses as the ones performed for the main study.

## Results

The mean age of the 53 initial participants was 22.1 years (SD 0.4) and 49% were males.

**Fig 1:**
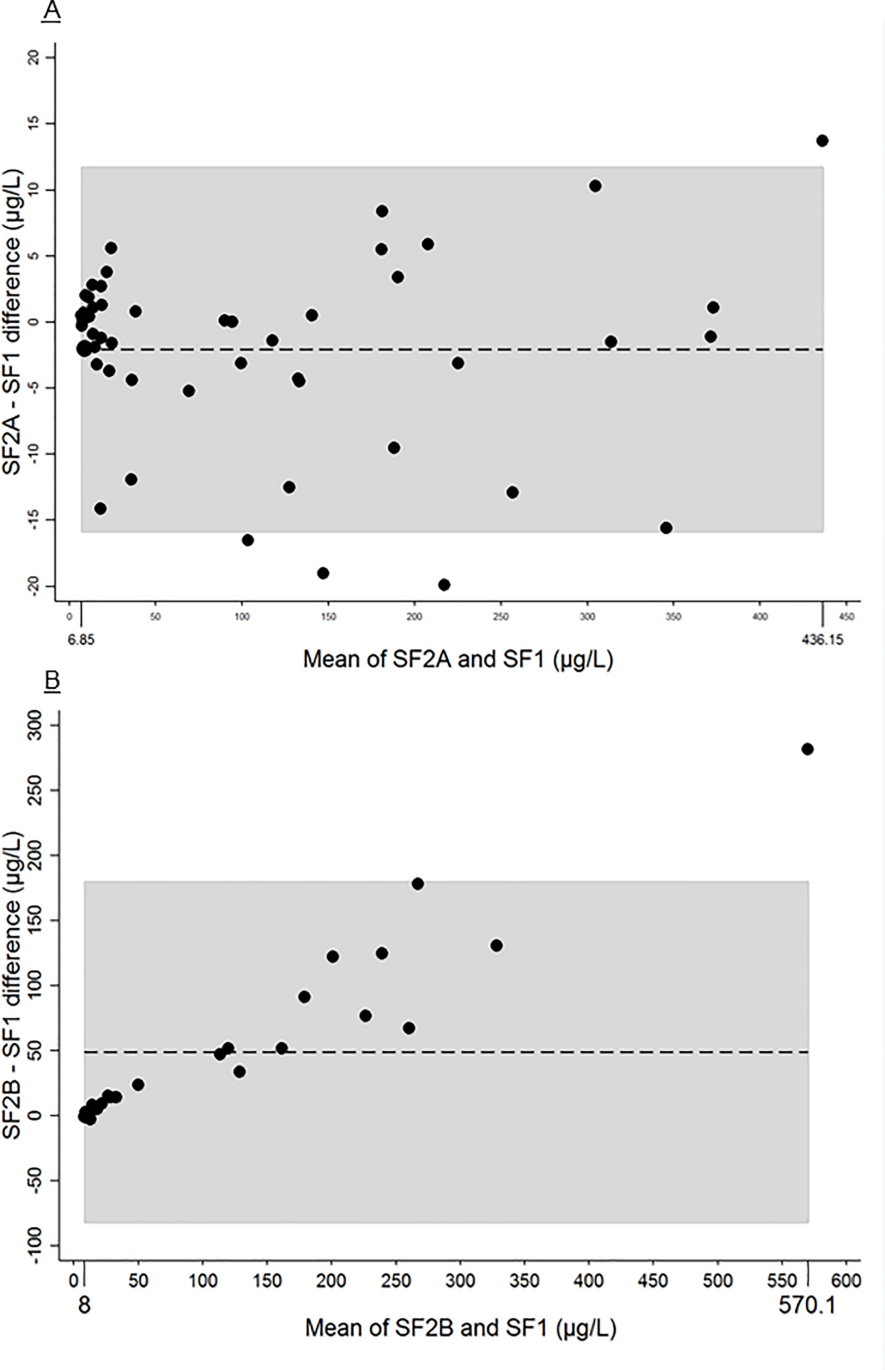
**A. Bland-Altman graph: mean of serum ferritin (SF) at the first (SF_1_) and second measure without intermediate thawing (SF_2A_).** Dotted line: mean difference between values of SF_1_ and SF_2A._ Grey zone: 95% limits of agreement for mean difference between SF_1_ and SF_2A._ **pB. Bland-Altman graph: mean of serum ferritin (SF) at the first (SF_1_) and second measure with intermediate thawing (SF_2B_).** Dotted line: mean difference between values of SF_1_ and SF_2B._ Grey zone: 95% limits of agreement for mean difference between SF_1_ and SF_2B_.

There were 53 paired values SF_1_ and SF_2A_. The mean (SD, range) values for SF_1_ and SF_2A_ were 108.6 μg/L (117.6, 6.6–429.3) and 104.6 μg/L (117.2, 7.1–443), respectively. The mean (SD) SF_2A_–SF_1_ difference was -2.1 μg/L (7.0, CV 2%). The equality line graph for SF_2A_ versus SF_1_ is in **S2 Fig**. The P-value from the *t* test for paired samples was 0.04; the ICC was 0.998 (95% confidence interval [CI] 0.997–0.999); and the Bland and Altman 95% LOA was -15.8 to 11.8 (**Fig 1A**). The ID proportions were 25% (95% CI 15–38) and 32% (95% CI 21–46) for SF_1_ and SF_2A_, respectively (mean difference in proportion SF_2A_–SF_1_: 8%, 95% CI 3–19; p_McNemar test_ = 0.046).

There were 28 paired values for SF_1_ and SF_2B._ The mean (SD, range) value for SF_2B_ was 135.2 μg/L (166.6, 7.5–710.9). The mean (SD) SF_2B_–SF_1_ difference was 48.9 μg/L (66.9, CV 30%). The equality line graph of SF_2B_ vs SF_1_ is in **S2 Fig.** The P-value of the *t* test for paired samples was < 0.001; the ICC was 0.88 (95% CI 0.76–0.94); and the Bland and Altman 95% LOA was –82.2 to 179.9 (**Fig 1B**). Among these 28 paired values, ID proportions were 32% (95% CI 17–52) and 25% (95% CI 12–45) for SF_1_ and SF_2B_, respectively (mean difference in proportion SF_2B_–SF_1_: -7%, 95% CI -26 to -2; p_McNemar test_ = 0.16).

The regression model found a strong interaction of thawing with SF_2_ in the relationship between SF_1_ and SF_2_ (P-value for the interaction term <0.001). Estimates of the model were SF_1_ = 4.38 + 0.99xSF_2_ without intermediate thawing and SF_1_ = 3.96 + 0.61xSF_2_ with intermediate thawing. The ICC between SF_1_, SF_2A_ and SF_2B_ in a two-way mixed-effects model (n = 28) was 0.91 (95% CI 0.84–0.95).

In post-hoc supplementary analyses, the participants’ mean age was 27.0 years (SD 0.1). The mean (SD, range) values for SF_A_, SF_B_, SF_C_ and SF_D_ were 124.8 μg/L (121.1, 34.9–363.2), 124.0 μg/L (121.9, 31.9–363.4), 122.6 μg/L (120.8, 31–359.9), and 123.1 μg/L (121.7, 30–362.5), respectively. The mean (SD) differences values for SF_B_-SF_A_, SF_C_-SF_A_ and SF_D_-SF_A_ were -0.8 μg/L (1.8), -2.2 μg/L (1.7), and -1.6 μg/L (2.3), respectively, and the Bland and Altman 95% LOA were -4.3 to 2.7, -5.5 to 1.1, and -6.1 to 2.8, respectively.

## Discussion

In this first evaluation of the comparability of SF levels in the long term, the agreement between immediate measures and those after freezing at -80°C varied to a limited extent, with a mean (SD) difference of -2.1 μg/L (7.0, CV 2%), ICC of 0.998 (95% CI 0.997–0.999), and Bland and Altman 95% LOA from -15.8 to 11.8 μg/L. Our results are consistent with previous reports of the comparability of SF levels after storage at -20°C, -70°C, -80°C and -196°C during shorter periods [18–20]. The mean difference observed in our study is much smaller than variations observed in the only available study reporting comparability after storage for 2 years [25] (**S1 Table**). The lower comparability observed in this latter study may be due to differences in storage temperatures (-25°C vs -80°C in our study) and the study’s non-paired design [25].

The variation in SF levels reported here was statistically significant but may be considered acceptable as compared with the repeatability precision and the within-laboratory precision CV provided by the manufacturer (0.4% to 4.7% and 1.0% to 4.9%, respectively) and the CV observed in a national quality insurance study that analyzed 2391 French private laboratories: 12.7% and 8.9% around 50 and 320 μg/L, respectively [31]. The absolute value of the mean difference, -2.1 μg/L, could have more impact when ferritin is dichotomized around low thresholds to study iron depletion than in studies of iron overload. Indeed, in our study, the iron depletion/deficiency proportion after long-term constant freezing was modified from 25% (13/53) to 32% (17/53) without intermediate thawing vs. from 32% (9/28) to 25% (7/28) with intermediate thawing. In the first case, 4 participants were reclassified as having ID according to SF_2A_ dosage and in the second case, 2 participants were reclassified as not having ID according to SF_2B_ dosage. These results suggests that when using SF measures from biobanks stored by freezing during several years, one should be cautious about diagnosis of ID.

Intermediate thawing had a major impact on the SF levels comparability after long-term storage by freezing, with a mean difference of 48.9 μg/L. The effect of thawing was greater with higher values of SF, as highlighted by the strong interaction identified in the regression model. Thus, based on these results, the use of SF levels measures after long-term freezing at -80°C and intermediate thawing should be discouraged.

The strengths of the present work are that all analyses were performed with the same technique in the same laboratory, with standardized protocols for storage, freezing and thawing, with a large range of SF values usually encountered in clinical practice (from 6.6 to 429.3 μg/L). We used several recommended statistical approaches to study the reproducibility of measurements [26, 32]. The design used did not allow for comparing variations in SF levels after long-term freezing to those observed during a short-time freezing period. However, in our post-hoc supplementary analyses, we compared SF measures on a convenience sample of sera of 6 participants after short-term freezing (seven days) with and without intermediate thawing. The variations observed in SF levels were not clinically significant, with or without intermediate thawing (for initial SF>30 μg/L). This suggests that intermediate thawing in the case of short-term freezing may not affect ferritin measures. This is consistent with the results reported by Gonzales-Gross et al. and Brinc et al. [22, 23]. Gonzales-Gross found that the mean difference in SF measures on aliquots frozen at -20°C continuously during 20 days after blood extraction vs with discontinuous freezing due to transport was -0.82 μg/L (95% CI -8.5 to +7.5) [22]. Brinc found that the variation in SF measures in aliquots kept frozen at -80°C during 13 months with a monthly freeze-thaw cycle before analysis were not significant (percentage change from baseline: ±9) [23].

An important limitation of our study is its retrospective design that did not allow for evaluating the variations in SF by repeated measures without freezing or by a replication study (with storage by short-term and long-term freezing, with or without intermediate thawing). Some hypotheses can be proposed to explain SF variations. Precipitation and desiccation are possible but unlikely, given the use of a standard procedure such as the Vortex system and the sealing of tube joints.

In conclusion, the agreement between SF values before and after 3 to 5 years of constant freezing at -80°C was in a generally accepted range, which supports the hypothesis of ferritin’s stability at this temperature for a long period. In long-term storage by freezing, intermediate thawing induced a major increase in values.

## Supporting information

S1 Dataset Dataset for our main analyses. (XLSX)

S2 Dataset Dataset for our post-hoc supplementary analyses. (XLSX)

S1 Fig Flow-chart. (TIF)

S2 Fig Equality line between the first serum ferritin measure (SF_1_) and the second measure without intermediate thawing (SF_2A_) or with intermediate thawing (SF_2B_). Full circles: values for SF_2A_ according to SF_1_ values (n = 53) with the equality line (full line). Hollow circles: values for SF_2B_ according to SF_1_ values (n = 28) with the equality line (dashed line).
(TIF)

S1 Table Comparability of biomarker levels used for iron deficiency diagnosis in the literature after various storage modalities. (DOCX)
