# Supplementary figures and images for: Stability of serum ferritin measured by immunoturbidimetric assay after storage at -80°C for several years
Source: PLoS One. 2017 Dec 11;12(12):e0188332. doi: 10.1371/journal.pone.0188332 (PMC5724861; doi:10.1371/journal.pone.0188332)

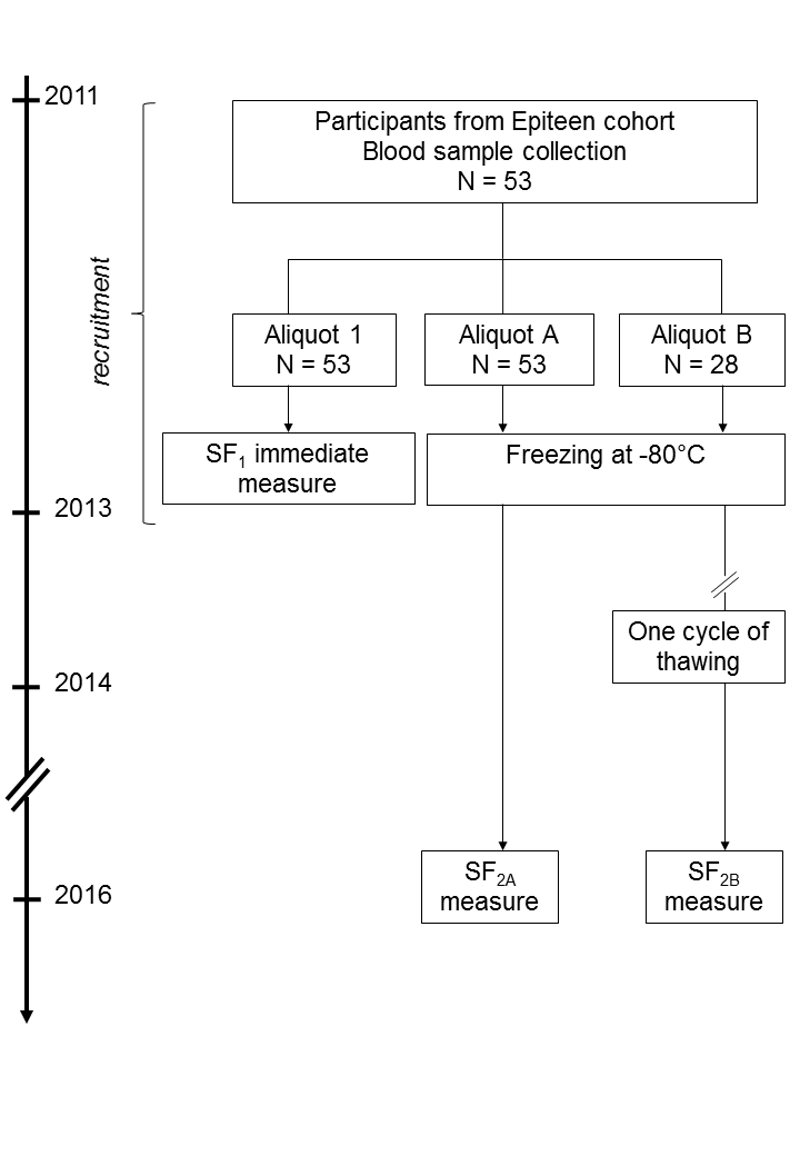

Supplement: S1 Fig — (TIF) [file pone.0188332.s003.tif]

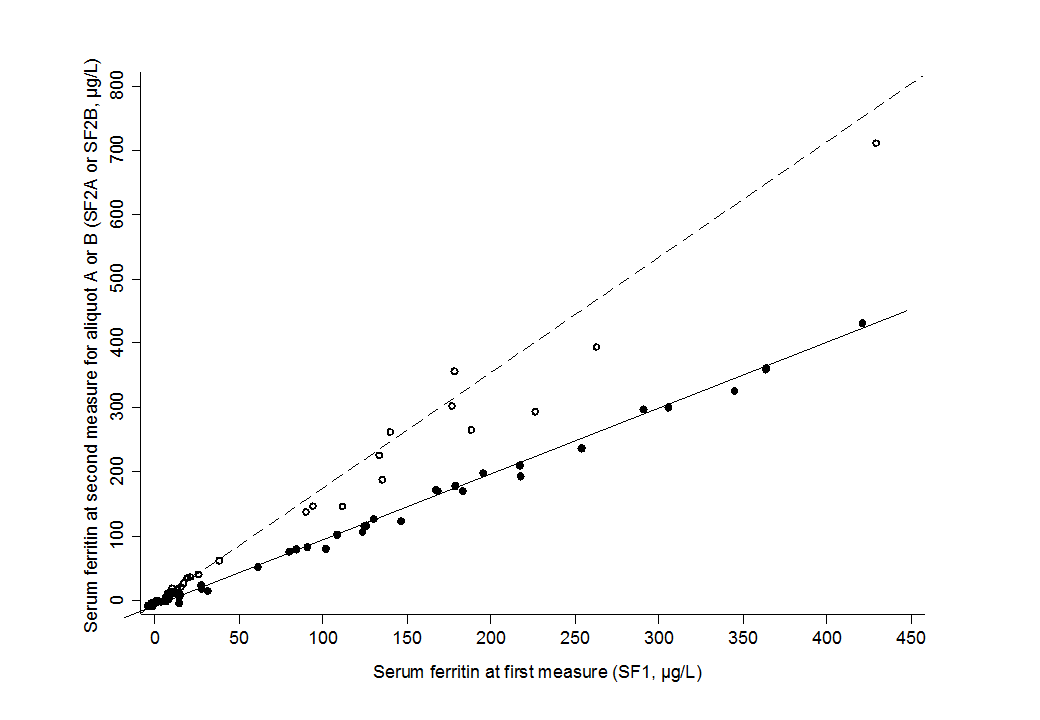

Supplement: S2 Fig — Full circles: values for SF2A according to SF1 values (n = 53) with the equality line (full line). Hollow circles: values for SF2B according to SF1 values (n = 28) with the equality line (dashed line). (TIF) [file pone.0188332.s004.tif]
