# Supplementary material for: Stability of serum ferritin measured by immunoturbidimetric assay after storage at -80°C for several years
Source: PLoS One. 2017 Dec 11;12(12):e0188332. doi: 10.1371/journal.pone.0188332 (PMC5724861; doi:10.1371/journal.pone.0188332)
Supplement: S1 Table — (DOCX) [file pone.0188332.s005.docx]

**S1 Table. Comparability of biomarker levels used for iron deficiency diagnosis in the literature after various storage modalities**

| **Temperature of storage** | **Duration of storage** | **Coefficients of variation (CV)** | **References** |
| --- | --- | --- | --- |
| **C-reactive protein level** | | | |
| -80 to -70°C | 7–14 years | < 10% | [[12](https://d.docs.live.net/3539d030e10f0334/Documents/CARMA/CARMA%20V_%20Stab%20ferritinemie/ARTICLE/PLOS%20One/Revision/Révision%20soumise/Supportingdata%20with%20Track%20Changes.docx#_ENREF_12)] [[13](https://d.docs.live.net/3539d030e10f0334/Documents/CARMA/CARMA%20V_%20Stab%20ferritinemie/ARTICLE/PLOS%20One/Revision/Révision%20soumise/Supportingdata%20with%20Track%20Changes.docx#_ENREF_13)] [[14](https://d.docs.live.net/3539d030e10f0334/Documents/CARMA/CARMA%20V_%20Stab%20ferritinemie/ARTICLE/PLOS%20One/Revision/Révision%20soumise/Supportingdata%20with%20Track%20Changes.docx#_ENREF_14)] |
| **Serum ferritin level** | | | |
| Positive temperatures | 4 hrs–15 days | 1–22% | [[15](https://d.docs.live.net/3539d030e10f0334/Documents/CARMA/CARMA%20V_%20Stab%20ferritinemie/ARTICLE/PLOS%20One/Revision/Révision%20soumise/Supportingdata%20with%20Track%20Changes.docx#_ENREF_15)] [[16](https://d.docs.live.net/3539d030e10f0334/Documents/CARMA/CARMA%20V_%20Stab%20ferritinemie/ARTICLE/PLOS%20One/Revision/Révision%20soumise/Supportingdata%20with%20Track%20Changes.docx#_ENREF_16)] [[17](https://d.docs.live.net/3539d030e10f0334/Documents/CARMA/CARMA%20V_%20Stab%20ferritinemie/ARTICLE/PLOS%20One/Revision/Révision%20soumise/Supportingdata%20with%20Track%20Changes.docx#_ENREF_17)] |
| -80°C | 4 days | 3% | [[18](https://d.docs.live.net/3539d030e10f0334/Documents/CARMA/CARMA%20V_%20Stab%20ferritinemie/ARTICLE/PLOS%20One/Revision/Révision%20soumise/Supportingdata%20with%20Track%20Changes.docx#_ENREF_18)] |
|  | 12 months | 2% | [[20](https://d.docs.live.net/3539d030e10f0334/Documents/CARMA/CARMA%20V_%20Stab%20ferritinemie/ARTICLE/PLOS%20One/Revision/Révision%20soumise/Supportingdata%20with%20Track%20Changes.docx#_ENREF_20)] |
| -196°C, -70°C and -20°C | 6 months | < 8% | [[19](https://d.docs.live.net/3539d030e10f0334/Documents/CARMA/CARMA%20V_%20Stab%20ferritinemie/ARTICLE/PLOS%20One/Revision/Révision%20soumise/Supportingdata%20with%20Track%20Changes.docx#_ENREF_19)] |
| -25°C | 2 years | -12.1% * | [[25](https://d.docs.live.net/3539d030e10f0334/Documents/CARMA/CARMA%20V_%20Stab%20ferritinemie/ARTICLE/PLOS%20One/Revision/Révision%20soumise/Supportingdata%20with%20Track%20Changes.docx#_ENREF_25)] |
|  | 25 years | -18.5% * | [[25](https://d.docs.live.net/3539d030e10f0334/Documents/CARMA/CARMA%20V_%20Stab%20ferritinemie/ARTICLE/PLOS%20One/Revision/Révision%20soumise/Supportingdata%20with%20Track%20Changes.docx#_ENREF_25)] |

* Percent difference of mean SF level in non-paired samples
